# Environmental effects of a management method used after fire on development of temperate Scots pine ecosystem: a 15-year study from Poland

**DOI:** 10.1007/s00267-023-01843-8

**Published:** 2023-06-09

**Authors:** Piotr Sewerniak, Maciej Markiewicz, Patrycja Tarnawska, Marta Wójcik

**Affiliations:** 1 Department of Soil Science and Landscape Management, Nicolaus Copernicus University in Toruń, Lwowska 1, 87-100 Toruń, Poland; 2 Department of Environmental Microbiology and Biotechnology, Nicolaus Copernicus University in Toruń, Lwowska 1, 87-100 Toruń, Poland

**Keywords:** Forest fire, Disturbance, Post-fire restoration, Soil preparation, Sandy soils, *Pinus sylvestris*

## Abstract

Due to the ongoing climate changes, temperate forests are increasingly exposed to fires. However, until now the functioning of post-fire temperate forest ecosystems with regard to used forest management method has been weakly recognized. Here, we examined three variants of forest restoration after fire (two variants of natural regeneration with no soil preparation—NR, and artificial restoration by planting following soil preparation—AR) regarding their environmental consequences in development of post-fire Scots pine (*Pinus sylvestris*) ecosystem. The study was conducted using a 15-year timespan in a long-term research site located in the Cierpiszewo area (N Poland) being one of the biggest post-fire grounds in European temperate forests in last decades. We focused on soil and microclimatic variables as well as on growth dynamics of post-fire pines generation. We found that the restoration rates of soil organic matter, carbon and most studied nutritional elements stocks were higher in NR plots than in AR. This could be primarily linked to the higher (*p* < 0.05) density of pines in naturally regenerated plots, and the subsequent faster organic horizon reconstruction after fire. The difference in tree density also involved regular differences in air and soil temperature among plots: consistently higher in AR than in both NR plots. In turn, lower water uptake by trees in AR implied that soil moisture was constantly the highest in this plot. Our study delivers strong arguments to pay more attention to restore post-fire forest areas with the use of natural regeneration with no soil preparation.

## Introduction

Fire is a disturbance which widely impacts the environment, with its effects on, i.a., atmospheric chemistry, forest functioning, carbon cycling, and land-use change (Keeley 2009; Lasslop et al. 2019,2020; Bullock et al. 2020; Hao et al. 2022). Burned woodlands constitute distinctly fire-altered grounds, with serious consequences in changes occurring in microclimate (Wolf et al. 2021) and soil properties (Neary et al. 1999; Certini 2005; Mayer et al. 2020). For example, post-fire forest areas are more exposed to insolation, which entails both increased soil organic matter mineralization and accordingly the loss of carbon and nutrients, as well as increased evaporation with its consequences in a forest site moisture conditions (DeBano et al. 1998; Shakesby and Doerr 2006; Mataix-Solera et al. 2011; Inbar et al. 2014). These changes are crucial for the existence and development of the re-established post-fire forest generation, and consequently practical foresters face the problem how to manage fire-affected woodlands to be vital and productive after such disturbance. Dilemmas being of especially high importance during the introduction of the post-fire forest generation refer to the choice of a soil preparation method used before the introduction of trees, as well as a method used for the forest regeneration. Regarding the former issue, site preparation methods of lower disturbance to soil are usually recommended (Aleksandrowicz-Trzcińska et al. 2014; Sewerniak et al. 2017; Chaves Cardoso et al. 2020), because the loss of soil carbon and nutrients increases with the intensity of soil scarification (Mallik and Hu 1997; Mayer et al. 2020; Zhang et al. 2021). However, in several European countries (e.g. Poland) the main soil preparation method which is still applied before introducing subsequent trees generation is a highly soil destructive method with the use of a forest plough (Sewerniak et al. 2012; Aleksandrowicz-Trzcińska et al. 2014). In turn, the dilemma regarding the regeneration method pertains to the two approaches, mainly: the usage of natural regeneration based on natural encroachment of trees, or artificial regeneration done usually by planting, which both have their inherent advantages and disadvantages (Barnett and Baker 1991; Löf et al. 2012).

It has been highlighted in many studies, that the projected change in temperature and precipitation (Knutti and Sedláček 2013) is expected to increase the risk of wildfires in European forests (Schelhaas et al. 2003; Szczygieł et al. 2009; Zell and Hanewinkel 2015). However, until recently, most of papers referring to the environmental effects of fire, as well as to management of post-fire areas in Europe have been focused on Mediterranean (e.g. de las Heras et al. 2012; Inbar et al. 2014) and boreal ecosystems (e.g. Kirdyanov et al. 2020; Zhao et al. 2021). The main indicated reason for this is the fact that these ecosystems are composed of plants with highly flammable compounds, and consequently fire disturbance is indicated as an integral part of natural dynamics of the ecosystems (Adámek et al. 2018; Feurdean et al. 2019). In turn, in temperate zone of Central Europe the role of fire in the functioning of woodlands has been traditionally marginalized (Tinner et al. 2005; Adámek et al. 2018). However, especially due to a projected increase in the frequency of heat and drought occurring in growing season (Knutti and Sedlacek 2013; Ciais et al. 2005), nowadays, the risk of the extreme events occurrence in temperate forests has clearly increased (Zell and Hanewinkel 2015), which is also highlighted directly to forest fires with reference to different regions of the globe (Schelhaas et al. 2003; Tran et al. 2020; Masinda et al. 2022; Jahdi et al. 2023). This trend has already been recognized based on the analysis of fire frequency in temperate European forests for the period 2009–2018 (Fernandez-Anez et al. 2021).

Regarding the increasing risk of fire occurrence, the considerable circumstance is that in extensive areas of Central and Eastern Europe conifer monocultures have been artificially introduced in areas being primarily overgrown with mixed or broadleaved forests (Zerbe 2002; Sewerniak 2020; Sewerniak and Jankowski 2021). Consequently, the woodlands became much more exposed to the fire risk, as conifer stands are much more susceptible to hazards (i.a. fires) than broadleaved stands (Kenk and Guehne 2001; Szczygieł et al. 2009). Among forest tree species, an especially high risk of fire has been highlighted for Scots pine (*Pinus sylvestris* L.) stands (Marozas et al. 2007; Niklasson et al. 2010; Adámek et al. 2018), which predominate in some countries of Central and Eastern Europe. For example, the share of the species in Polish forests equals almost 60% (Statistics Poland 2022). Hence, the recognition of the post-fire development of Scots pine ecosystem with regard to temperate zone is of a high importance both from the scientific point of view and from the land management perspective.

The lack of hitherto detail knowledge in this field, as well as the increasing importance of the problem following the ongoing climate changes, encouraged us to investigate the post-fire development of Scots pine ecosystems regarding the two key forest management topics: soil preparation before trees introduction and forest regeneration method (natural by seeding vs. artificial by planting). Hence, the main aim of this study was to examine potential differences in post-fire Scots pine ecosystems referring to microclimate, soil properties and growth dynamics of newly established pine generation, being caused by different management methods used after fire. Additionally, following the fact that the study has been conducted in a long-term research area including a 15-year timespan, here, we also aimed to show how the examined variables evolved at the early development stage of pine ecosystems. Thus, in this study we tried to answer the following questions: (1) How does a management method used after fire (natural forest regeneration with no soil preparation vs. artificial regeneration by planting following soil preparation) affect the studied characteristics of Scots pine ecosystem? and (2) How do the characteristics change at early stage of post-fire pine ecosystem development?

## Materials and methods

### Study area and the investigated plots

The study was conducted in the Cierpiszewo post-fire area (N Poland, ca. 3000 ha of pine stands burned in 1992) being one of the biggest burned areas of temperate European forest in last decades. The area covers river terraces built of loose sand, which, in some parts, was relocated by aeolian processes forming inland dunes. The study site is characterized by transitional climate between oceanic and continental. The average annual temperature is 7.9 °C, and mean annual precipitation equals 522.5 mm, with July as the wettest month. Monthly averages of temperature range from −2.2 °C in January to 18.1 °C in July, and the average length of the annual growing season equals 218 days (Wójcik and Marciniak 2006).

**Fig. 1:**
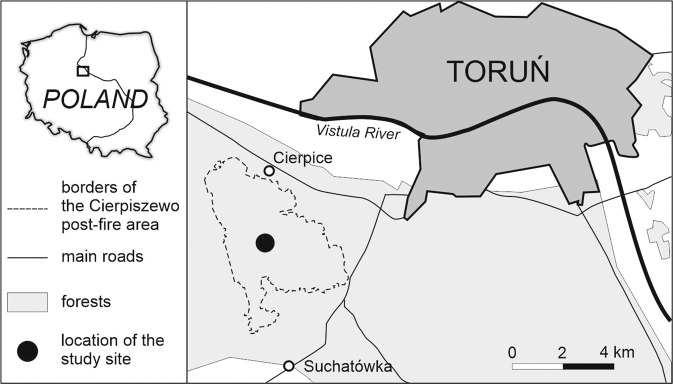
Location of the study site

**Fig. 2:**
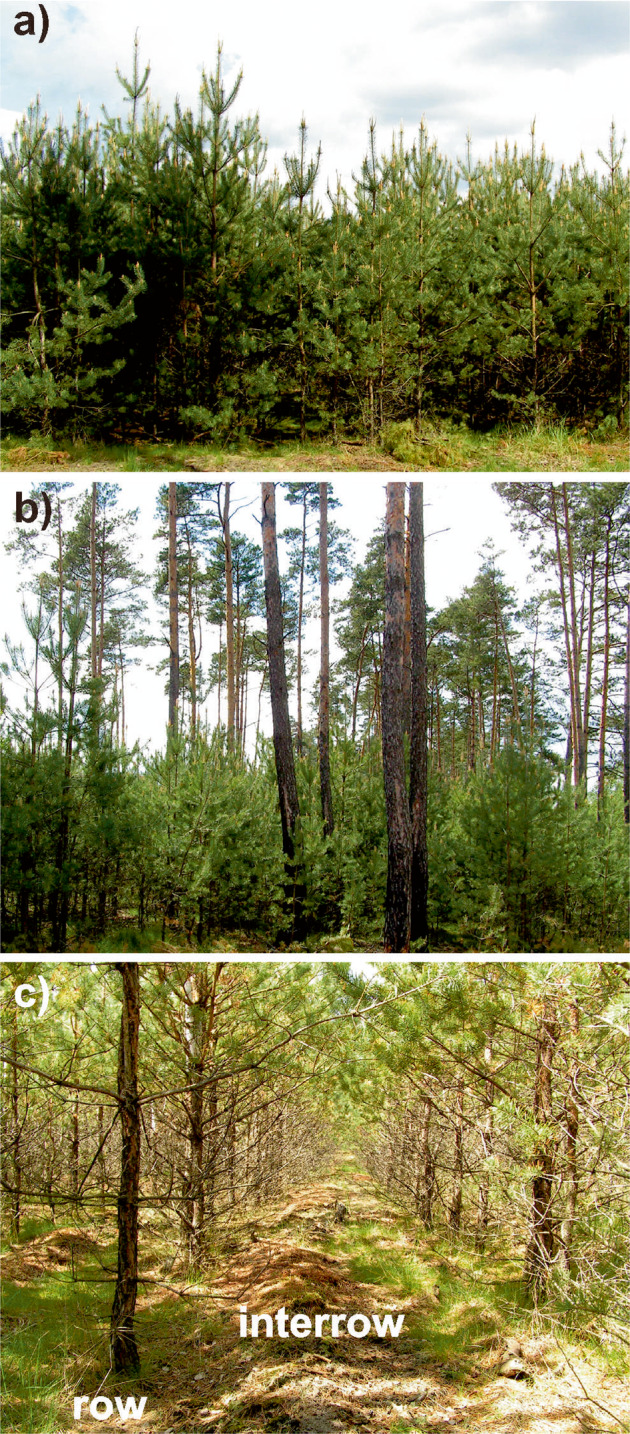
Studied plots (2006): (**a**) a naturally regenerated stand based on seeds from adjacent maturing pine forest which survived the fire in 1992 (NR), (**b**) a naturally regenerated stand occurring under canopy of maturing pines which survived the fire in 1992 (NR-UC), (**c**) an artificially regenerated stand by planting with a soil preparation before the planting using a mouldboard plough (AR)

The study plots were established in 2006 in pure Scots pine forest located in the central part of the Cierpiszewo area (52°57’04”N, 18°27’33”E, Fig. 1). The research was conducted using a 15-year timespan (2006–2021). The examined plots represent 3 variants of management method used after the fire in 1992 (Fig. 2): (1) a naturally regenerated stand based on wind dispersal of seeds from adjacent maturing pine forest which survived the fire; no soil preparation method was used after the fire (NR), (2) a naturally regenerated stand with post-fire pines occurring under a canopy of the maturing pines in upper storey which survived the fire (a canopy cover of the upper storey equals 30%); no soil preparation method was used after the fire (NR-UC), (3) an artificially regenerated stand after the fire by introducing young pines by planting; the soil was prepared before the planting using a mouldboard forest plough (AR). The post-fire pines occurring in all plots were at the same age in all the plots (13 years in 2006). In both naturally regenerated stands no cuttings have been executed, while they were two times carried out in AR due to the regulations according to the operative forest management plans. All the plots were the same in size (0.24 ha), and located in close vicinity to one another (within ca. 100 m). Consequently, the pedological backgrounds of the plots have been the same. They were represented by acidic Brunic Arenosol (IUSS Working Group WRB 2022) formed from loose fluvial sand in a flat river terrace.

### Data collection

The research was initiated with tree measurements in 2006, which were subsequently repeated in 2021. A canopy cover of post-fire pine generation was 95% in both years in NR, 85 and 95% in NR-UC, 80 and 75% in AR (in 2006 and 2020, respectively). Parameters of pines representing the post-fire generation (height and diameter at the breast height, i.e., 1.3 metres above the ground level) in naturally regenerated plots (NR and NR-UC) were examined in regularly located 10m^2^ circular subplots in which all occurring trees were measured. The number of subplots was 24 for NR and NR-UC in 2006, as well as for NR-UC in 2021. While for NR the number of circular subplots was 12 in 2021. This was the case because a cutting was executed in external part of NR by foresters, which resulted in exclusion of that part of the plot from the research in all investigations done in 2021. Even so, the number of measured pines in NR in 2021 was relatively high (193 trees). Because of the fact that in AR pines were regularly planted in rows (Fig. 2), circle subplots were not applied in this site. Instead, in both investigation years all pines occurring in every second row within the plot were measured. Due to the fact that rows in which pines were planted were regularly located in AR, for statistical examinations of pines density the whole 0.24 ha area of the plot was divided by the number of rows. Subsequently, numbers of pines occurring in particular rows were divided by the area calculated for a row, and thus 10 replicates for analysis of pines density in AR were obtained. In total, in all the examined plots 1918 pines were surveyed in 2006 (NR: 495, NR-UC: 518, AR: 905), while they were 796 in 2021 (193, 295 and 308, respectively). Depending the tree size, a calibrated pole or a Vertex IV hypsometer (Haglöf, Sweden) was used for height measurements, while diameters were determined using a calliper. Raw data showing results of trees measurements are presented in Online Resource 1.

**Fig. 3:**
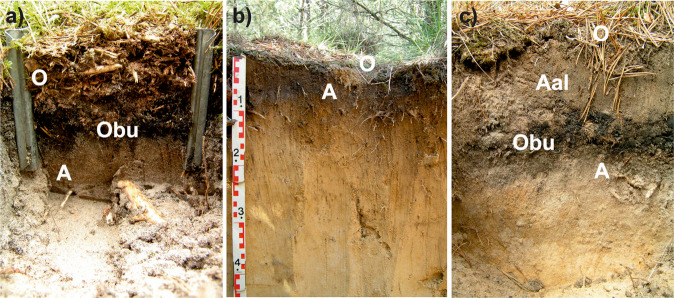
Soil morphology in a studied plots: (**a**) naturally regenerated stands (NR and NR-UC), (**b**) rows in an artificially regenerated stand with a soil prepared using a plough before the planting (AR-r), (**c**) interrows in artificially regenerated stand with a soil prepared using a plough before the planting (AR-ir)

The main pedological part of the study was conducted in 2008 and in 2020, when topsoil samples were collected for laboratory analyses. In naturally regenerated plots 3 horizons were examined: surface organic horizon being formed since the fire in 1992 (O), organic horizon partly burned during the fire (Obu), and humus horizon (A). In AR the soil research was done simultaneously in rows (AR-r) and in interrows (AR-ir), which, as a result of the soil preparation with a plough, differed regarding a topsoil morphology. Namely, Obu horizon was ploughed out before the planting and consequently absent in rows, while in interrows allochthonous humus horizon (Aal) occurred being placed in interrows during ploughing. To enable further calculation of bulk density and stocks, soil samples in each plot were collected using 10 × 10 cm iron frame within which material of the investigated horizons was collected. Organic horizons (O and Obu) and Aal horizon were sampled covering the total horizon thickness, while the underlying primary (pre-fire) A horizon was collected in each plot with the constant thickness of 3 cm. In each of all 4 variants (NR, NR-UC, AR-r, AR-ir), soil samples were collected in 6 replicates in 2008, and in 5 replicates in 2020. Consequently, in total, 132 soil samples were examined in our study (72 collected in 2008, and 60 in 2020). The morphology of the examined topsoils is shown in Fig. 3.

The collected in both years (2008 and 2020) soil samples were air-dried, organic samples were mechanically ground, and mineral samples were sieved using a 2 mm mesh. Subsequently, the samples were subjected to the following determinations (Bednarek et al. 2004; IUSS Working Group WRB 2022): bulk density based on samples dry weight and the measured in a field horizons thicknesses; content of soil organic matter (OM) by combustion of a sample at 550 °C; total organic carbon (C) and total nitrogen (N) content by using a CHN/CHNS Macro Elemental Vario MACRO Cube analyzer; exchangeable nutritional basic cations (Ca^2+^, Mg^2+^, K^+^) contents after extraction with 1 M ammonium acetate at pH 7.0, by a SOLAR 969 atomic absorption spectrophotometer. All determinations were done using all replicates, with exception to the examination of Ca, Mg and K content in 2006. In the latter case a composite sample was formed from 6 replicates for each horizon in each plot, in which the contents of these elements were determined. Obtained subplot raw data regarding the soil characteristics are shown in Online Resource 2.

Additionally, dynamics of topsoil moisture and temperature were investigated in the examined plots in the 2013 growing season. Regarding the artificially revegetated plot this part of the study was done in rows (AR-r). The temperature was measured at a depth of 3 cm (in A horizon), while the moisture was examined at four depths of a mineral topsoil (3, 10, 25 and 50 cm). The parameters were surveyed from 5th April to 4th December 2013 with the interval of ca. 2 weeks (in total, the measurements were done in 18 days). In this examinations, soil moisture was determined using the TDR method, while soil temperature was measured using a digital thermometer. Due to relatively dry soils being investigated, in moisture measurements a TDR probe was calibrated for low soil water content examinations (Skierucha et al. 2008).

The microclimatic part of the study was conducted from 2009 to 2020 with the use of automatic HOBO loggers being installed, and shed against sunshine with bright cover, 30 cm above a ground level. The loggers were placed in the 3 studied plots, and measured air temperature and humidity continuously in the studied timespan with an interval of 1 h.

### Data analysis

Stocks of particular variables (OM, C and N given in kg·m^−2^, Ca^2+^, Mg^2+^ and, K^+^ given in mol·m^−2^) were calculated separately for all examined soil horizons as follows:

$$S = \left( {C\cdot D\cdot H} \right)/10$$

where S is a stock, C is a content of a variable, D is bulk density [g·cm^−3^], H is a thickness [cm] of a soil horizon. After the calculations, the stocks obtained for the horizons were additionally summed for all investigated topsoil horizons in each studied subplot being used for soil examinations.

Based on the TDR soil moisture measurements, soil water storage (SWS) was calculated for each plot in every of 18 measure days. This was done to the depth of 65 cm using the equation:

$$SWS = \left( {M\cdot H} \right)/10$$

where SWS is a soil water storage [mm] for a soil layer, M is a mean soil moisture [%] for depths of 5, 10, 25 or 50 cm, and H is a thickness of a soil layer representing particular depth of moisture measurements [cm]. After calculating SWS for the layers, the values obtained were summed for every measure day for each plot to the depth of 65 cm.

Many of the studied variables differed from the normal distribution (*p* values in the Shapiro-Wilk test <0.05). Hence, non-parametric tests were employed in the statistical analyses. The U Mann–Whitney test was used for the examination of difference between two samples, and the Kruskal-Wallis with post hoc Dunn’s tests were used to assess the significance of differences among more than 2 samples. The Friedman with post hoc Wilcoxon tests were applied to examine the significance of differences among the plots with regard to dynamics of SWS and soil temperature as well as to monthly averages of air temperature and relative air humidity. Detected differences were deemed significant if *p* < 0.05. The statistical analyses were conducted using PAST ver. 4.12 (Hammer et al. 2001). The numbers following the mean values in the text with ±symbols refer to standard errors.

The differences in pine attributes among the three studied plots and between the two examined years were analysed using the two way Scheirer Ray Hare test with R 4.3.0 software (R Core Team 2023). Subsequent pairwise comparison of individual combinations of year and plot variant was conducted in this software using the Dunn post hoc test.

## Results

### Soil properties

**Fig. 4:**
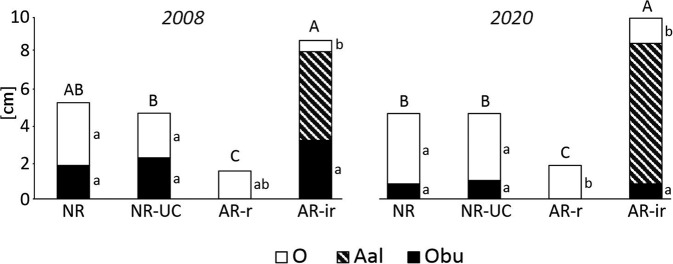
Mean thickness of topsoil horizons overlying the primary (pre-fire) A horizon in 2008 and 2020. O – surface organic horizon being formed in the post-fire pine stand generation; Obu – burned organic horizon in the fire in 1992; Aal – allochthonous humus horizon being ploughed out from rows before artificial forest regeneration and placed in interrows; NR natural regeneration, NR-UC natural regeneration under canopy, AR-r artificial regeneration (rows), AR-ir artificial regeneration (inter-rows). Different capital letters over bars indicate significant difference in a total topsoil horizons thickness among plots, while different lowercases at bars show significant difference in a thickness regarding a given soil horizon among plots

Regarding thickness of topsoil horizons, we found both differences among plots, as well as between the two years of the study within particular plots. When the total thickness of the examined topsoil horizons were considered, the significantly highest values comparing to other plots were found for AR-ir (9.5 ± 0.9 cm in 2008, and 10.8 ± 0.3 cm in 2020). However, this difference resulted from the fact that the allochthonous humus horizon Aal occurred only in AR-ir, while it was lacking in other studied soils (Figs. 3 and 4). In turn, the thickness of O horizon was the lowest in AR-ir, and for both studied positions in AR (rows and inter-rows) O horizon was smaller than in both naturally revegetated plots (Fig. 4). The differences stated in 2008 and 2020 for the thickness of Obu horizon among the examined plots were non-significant. However, the significant (*p* < 0.05) decrease in this variable between 2008 and 2020 for all the investigated plots was revealed (Fig. 4). The opposite trend was found for O horizon, which became thicker between 2008 and 2020, however, the change was only at the border of statistical importance for NR-UC and AR-ir (*p* = 0.05, and 0.07, respectively), while for NR and AR-r it was non-significant.

**Fig. 5:**
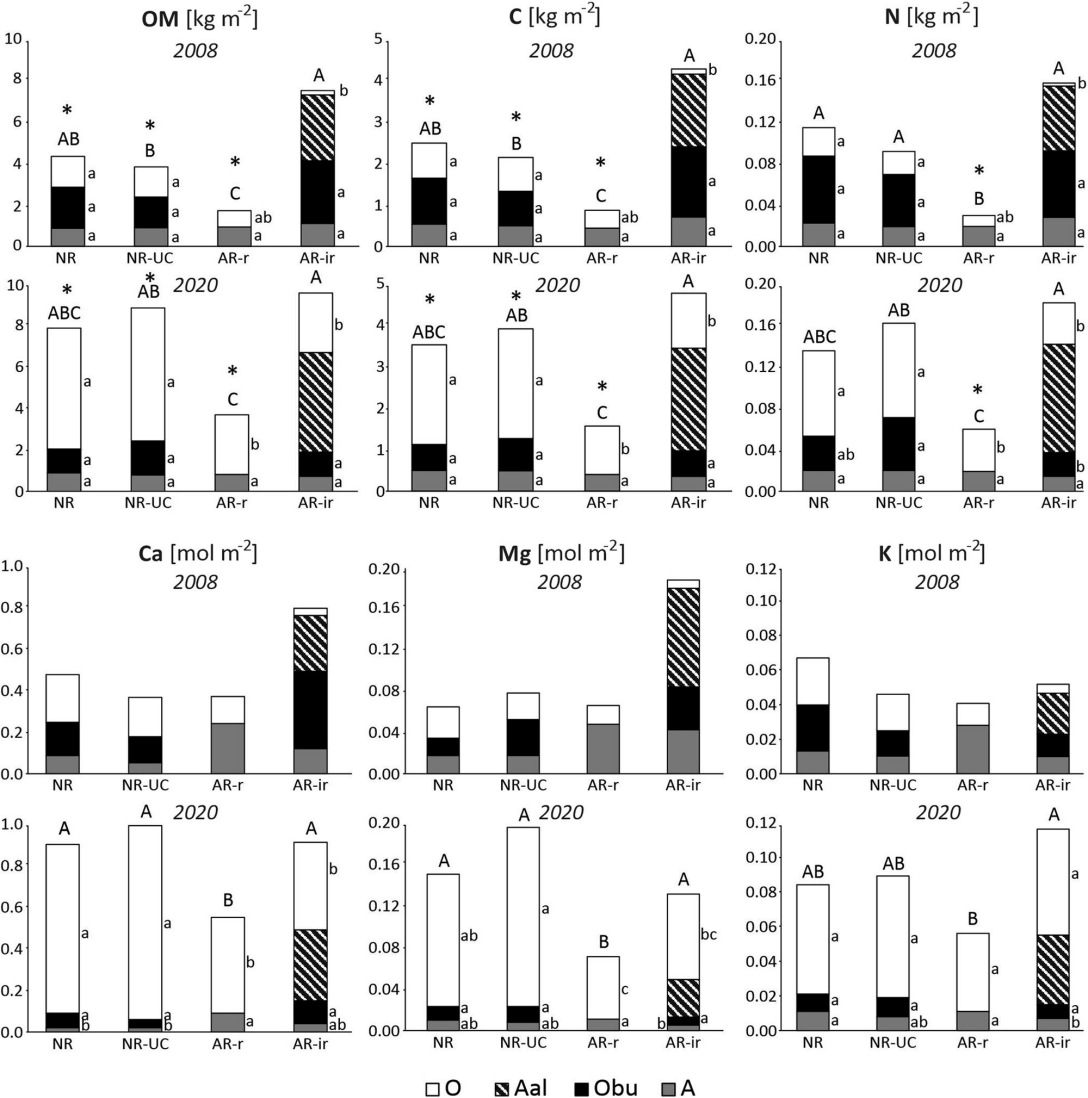
Topsoil mean stocks of soil organic matter (OM), organic carbon (C), total nitrogen (N), and exchangeable Ca, Mg, K in 2008 and 2020. NR – natural regeneration, NR-UC – natural regeneration under canopy, AR-r – artificial regeneration (rows), AR-ir – artificial regeneration (inter-rows). Different capital letters over bars indicate significant difference in total stocks among plots, while different lowercases at bars show significant difference in stocks regarding a soil horizon among plots. Asterix over a bar indicates a significant difference in total stocks between 2008 and 2020 in a given plot

The clear highest topsoil stocks of organic matter as well as the studied elements were usually found in AR-ir, while the lowest in AR-r. However, the difference among AR-ir and both naturally revegetated plots (NR and NR-UC) disappeared in the examined 12 years, and consequently existing significant differences found for 2008 became non-significant among the plots in 2020 (Fig. 5). In turn, the lowest total stocks revealed in AR-r in 2008 stayed mostly significant also in 2020 when compared to other plots (Fig. 5). As it can be seen from Fig. 4, the progress in stocks of OM and the examined elements between 2008 and 2020 resulted from the clear increase of the stocks in O horizon. In many cases the stocks increased in this horizon several times in the studied period. For example, the stocks of OM in NR enlarged between 2008 and 2020 almost 4 times (from 1.48 ± 0.25 in 2008 to 5.81 ± 0.53 kg m^−2^ in 2020, *p* < 0.05). The increase referring to the total stocks including records regarding all topsoil horizons was not so spectacular. This was caused by the decrease in the stocks stored in burned organic horizon (Obu) progressing with time (Fig. 5). When the total stocks are considered, the smallest progress between 2008 and 2020 was found regarding interrows in artificially revegetated plot. Compared to other plots, in AR-ir the total stocks increases were minor (with the only exception for potassium), and regarding the total magnesium stocks the decrease between 2008 and 2020 was even found in AR-ir, which was not revealed in any other plot (Fig. 5). Numerical data together with relevant SE values referring to thickness of topsoil horizons as well as to topsoil stocks are shown by horizons in Online Resource 3.

**Fig. 6:**
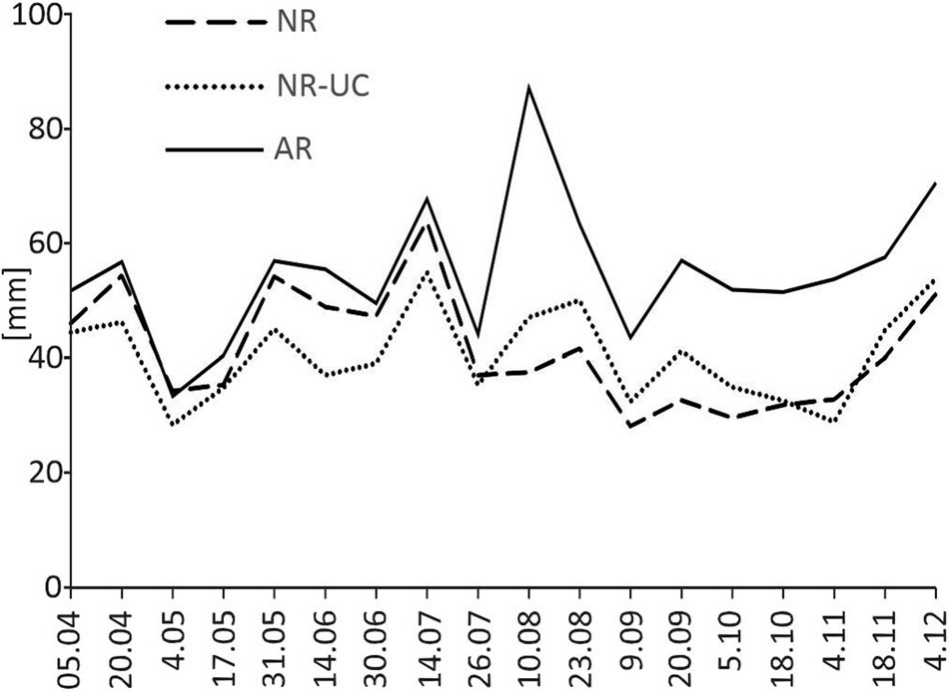
Dynamics of soil water storage summed to the soil depth of 65 cm for the 2013 growing season. NR natural regeneration, NR-UC natural regeneration under canopy, AR artificial regeneration (rows)

**Fig. 7:**
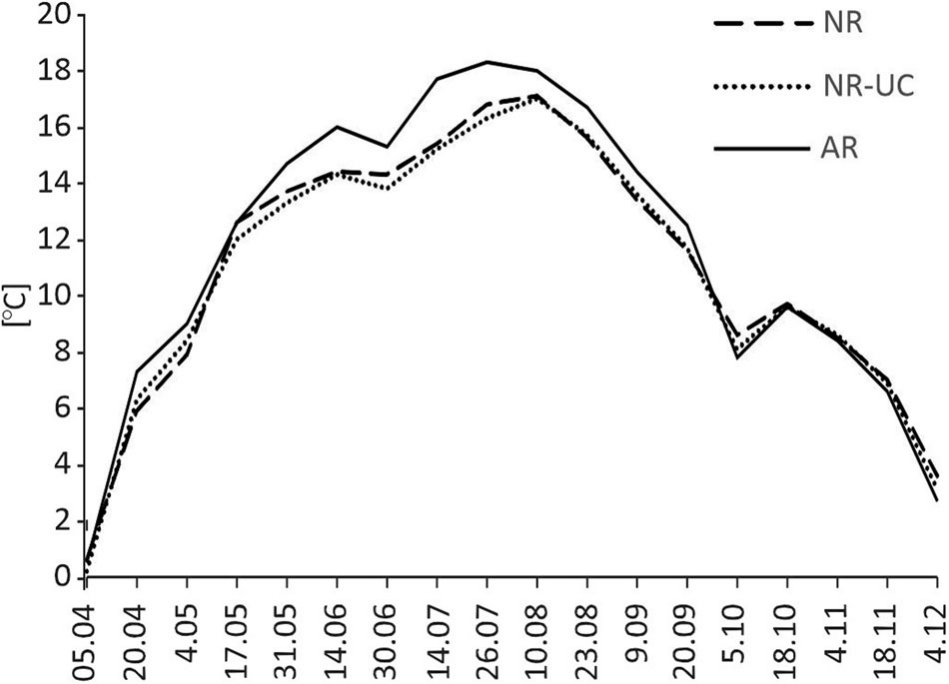
Dynamics of soil temperature at a depth of 3 cm for the 2013 growing season. NR natural regeneration, NR-UC natural regeneration under canopy, AR artificial regeneration (rows)

Very clear trends were found with regard to the differentiation of soil water storage and soil temperature dynamics among the studied plots. With regard to SWS the highest values were consistently revealed for AR, which involved significantly difference between AR and both remaining studied plots. While regarding the comparison between the naturally revegetated plots the relationship was determined by the part of a growing season. Specifically, in its first half (from April to July) the SWS values were constantly and thereby significantly (*p* < 0.05) higher in NR than in NR-UC, while in late summer and in autumn the difference between the plots was also statistically significant, but opposite (Fig. 6). Such pattern was not found with reference to the dynamics of soil temperature. Namely, in both naturally revegetated plots these dynamics were very similar within the entire examined period (Fig. 7). Regarding the soil temperature 3 cm below the ground level, the special trend was revealed for AR, in which the temperature in spring, and especially in summer was consequently the highest among other examined plots (Fig. 7). Hence, with regard to soil temperature AR significantly differed from both naturally revegetated plots.

### Microclimatic variables

Monthly average air temperatures showed the consistent trend for all months between March and October. The lowest means were found for NR, the middle for NR-UC, and the highest for AR, however, the only significant difference referred to the difference between NR and both remaining plots. In turn, regarding air humidity the clear opposite trend was revealed with the highest values found for NR, and the lowest for AR (Table 1), and all the differences among the plots were deemed as statistically significant (*p* < 0.05). These relationships are also reflected in yearly averages, which equalled 8.1 °C in NR, 8.3 °C in NR-UC, and 8,4 °C in AR regarding air temperature, and 86.9, 86.2, and 84,7%, in NR, NR-UC, and AR, respectively, with reference to air humidity (Table 1). The trends were of much minor importance for winter months, when, in general, the differences in the examined microclimatic variables among the studied sites were much smaller than those revealed for the growing season.

**Table 1:** Monthly averages of air temperature and relative air humidity 30 cm above the ground level (2009–2020) in the studied plots

Plot	I	II	III	IV	V	VI	VII	VIII	IX	X	XI	XII	Mean
Temperature [°C]													
NR	−2.3	−1.1	2.5	7.9	12.8	16.7	18.3	17.8	12.7	7.5	3.8	0.4	8.1
NR-UC	−2.1	−1.1	2.6	8.3	13.1	16.9	18.6	18.1	13.0	7.6	3.9	0.5	8.3
AR	−2.2	−1.1	2.7	8.4	13.2	17.1	18.8	18.2	13.1	7.6	3.8	0.5	8.4
Humidity [%]													
NR	94.8	92.4	86.9	76.5	78.0	78.9	82.1	81.4	86.6	92.0	96.7	96.8	86.9
NR-UC	95.1	92.5	85.9	74.8	76.8	78.0	80.8	80.2	85.6	91.8	96.4	96.8	86.2
AR	93.8	90.7	83.9	73.4	75.6	76.6	79.2	78.6	83.9	90.1	95.0	95.1	84.7

*NR* natural regeneration, *NR-UC* natural regeneration under canopy, *AR* artificial regeneration

### Growth dynamics of trees

**Fig. 8:**
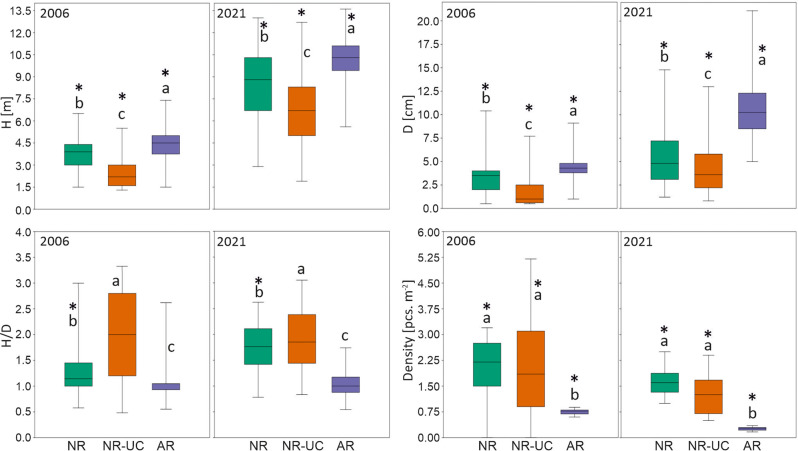
Pine attributes measured in the studied plots in 2006 and 2021. H – height, D – diameter, H/D – slenderness. Different lowercases at boxes show significant difference in a variable among plots in a given year. Asterix over a box indicates a significant difference in a variable between 2006 and 2021 in a given plot. NR natural regeneration, NR-UC natural regeneration under canopy, AR artificial regeneration

The investigated plots significantly differed regarding growth dynamics of re-established pines after the fire in 1992. In both studied years the highest dimensions of trees were revealed for AR, the middle values were found for NR, while the smallest pines occurred in the naturally revegetated plot under the canopy of older trees (NR-UC, Fig. 8). In turn, in both years the pines in NR-UC featured the highest slenderness, while those occurring in AR showed the lowest records among all the studied plots. All the differences regarding the pine height, diameter and slenderness were of significant importance (*p* < 0.05) among the examined plots (Fig. 8). As far as the density of trees is considered, the variable was several times lower in AR when compared to both naturally regenerated plots (Fig. 8). The importance of a management method used after fire for the studied pine attributes was also revealed in the two way Scheirer Ray Hare test. The test indicated also statistically significant effect of the year and of the interaction year x plot for the pine attributes. All the relations were found significant at *p* < 0.05 with the only exception of the interaction year x plot with reference to stand density (Table 2). Detailed results of pairwise comparison of individual combinations of year and plot variant were shown in Online Resource 4.

**Table 2:** Results of the two-way Scheirer Ray Hare test showing importance of year and plot for the studied pine attributes

	Df	H-test	*p* value
Height			
Year	1	1457.68	<0.001
Plot	2	509.73	<0.001
Year x Plot	2	19.90	<0.001
Diameter			
Year	1	616.62	<0.001
Plot	2	894.91	<0.001
Year x Plot	2	25.81	<0.001
Slenderness			
Year	1	36.31	<0.001
Plot	2	931.45	<0.001
Year x Plot	2	62.85	<0.001
Density of trees			
Year	1	8.217	0.004
Plot	2	30.9888	<0.001
Year x Plot	2	0.3604	0.83

## Discussion

### Effects of the management methods

Our study showed a clear effect of a management method used after fire on characteristics of temperate Scots pine ecosystem at an early stage of its development after a disturbance. The primary factors involving subsequent differences among the investigated plots were (i) the different tree density, and (ii) the application of soil preparation after fire. Young forest stands being naturally regenerated are usually much denser than stands artificially recovered by planting (Modrý et al. 2004; Jandl et al. 2007), which was clearly seen also in our study. Lower pines density in AR can explain higher air and soil temperature as well as lower air humidity, which all followed higher insolation caused by lower tree crowns cover (York et al. 2003; Gray et al. 2005). Apparently, this argumentation could be found as contradictory with the fact that SWS was revealed the highest in our study in AR. However, this can be explained by the lowest soil water uptake by pine roots following much lower tree density than in naturally revegetated plots. Thus, our study showed that regarding the trees density its relation with the water uptake can be more decisive for soil moisture than its consequences referring to ground insolation. The crucial importance of tree density on soil water uptake was highlighted in previous studies (McDowell et al. 2008). The lower tree density in AR implied also the occurrence of a thinner O horizon, which could also be linked to the found higher heating of mineral topsoil in this plot comparing to naturally revegetated plots. Specifically, organic soil material, especially by its low density, is a good thermal isolator, and consequently O horizon thickness is negatively correlated to heating of underlying mineral topsoil (Wojtkowiak et al. 2003; O’Donnell et al. 2009).

The differences in the pines densities can also explain the differences in trees slenderness among the plots. The higher values of the variable in both naturally regenerated stands than in AR could be simply linked to the higher density of trees, and consequently the higher competition to light among young pines. *Pinus sylvestris* has high demands for light (Bolibok and Andrzejczyk 2008), and similar relations between density and slenderness were reported in literature (Wang et al. 1998). In turn, the found in our research higher H/D of pines examined in NR-UC than in NR can also be explained by the light factor as the former trees have grown under a canopy of older, pre-fire pines. Consequently, they are less exposed to sunlight and must compete more for light to survive than pines in NR. The occurrence of the canopy of older trees can likely also explain the found pattern of SWS dynamics among both investigated naturally recovered plots. In growing season, the needs of pines for soil water uptake are the highest in its first part (Vincke and Thiry 2008). Thus, the decreased SWS values found for spring and the turn of spring and summer in NR-UC can be linked to the occurrence, and thus the increased soil water uptake, by the pines occurring in the upper storey in this plot.

Soil preparation is a commonly used practice in forestry before the introduction of a new forest generation (Löf et al. 2012; Meyer and Ammer 2022). This is done to improve the existence conditions of a newly established forest stand generation (Aleksandrowicz-Trzcińska et al. 2014; Węgiel et al. 2023). However, soil preparation disrupts natural morphology of a forest soil (Löf et al. 2012; Ramantswana et al. 2020), and, by the increase of soil organic matter exposition for oxidation and the increased temperature, is supposed to support carbon and nutrients emission from a soil (Kirschbaum 2004; Zhang et al. 2021). Consequently, foresters are recommended to use soil preparation methods of minor effect on a soil rather than those which affect pedons aggressively (Sewerniak et al. 2017; Mayer et al. 2020). Besides, in sustainable forestry it is recommended to, wherever possible, introduce a new forest generation with no soil preparation using natural regeneration, mainly (Crouzeilles et al. 2020). Our study supported such attitude by showing advantages of natural regeneration with no soil scarification vs. artificial trees introduction by planting following a soil preparation. Although pines in naturally regenerated plots were on average smaller comparing to pines occurring in AR, this could be in a big part explained by selective cuttings executed by foresters twice in the latter plot which was not done in NR and NR-UC. Predominant in height and thus being exposed for insolation pines in both naturally regenerated plots showed high growth dynamics and surely can form robust skeleton of future mature stand in the plots. High vitality of naturally regenerated pines was highlighted also for other extensive burned forest areas in Central Europe (Hille and Ouden 2004; Dobrowolska 2008). Additionally, the high density of trees occurring in naturally regenerated sites diminishes the risk of deer serious damages occurrence in forest stands by dispersing the pressure of those ungulates in a relatively high number of pines (Miścicki et al. 2002; Pfeffer et al. 2021).

### Effect of time

Carbon sequestration in a soil is strictly and positively related to vegetation biomass (Yuan et al. 2022). The found in our study increase in stocks of SOM and of particular elements is typical for the development of young forest stands, and is caused mainly by the increasing with stand age alimentation of tree litterfall to a topsoil (Grüneberg et al. 2014; Jasińska et al. 2019). However, our study showed that the increase was strongly spatially differentiated when a soil is prepared with a forest plough before artificial stand regeneration by planting. After primarily concentration of soil organic carbon and nutrients in interrows, they are both more effectively stored in rows. On the one hand, this can be caused by the lower micro-topographical location of the rows and thus gravitational concentration of soil organic matter and nutrients in this position. The concentration can be additionally supported by the fact that young trees delivering litterfall are planted in rows, and their branches with needles occur primarily over the rows, which is relevant especially for several initial years after the planting. On the other hand, the soil material in interrows, primarily due to the initially lower shadowing by young trees and consequently higher insolation, is more exposed to organic matter mineralization, which implies the loss of organic carbon and nutrients (Zhang et al. 2021; Li et al. 2023). This is especially crucial for the functioning of woodlands with sandy soils, in which organic matter constitutes the main available source of nutrients to plants (Elgersma 1998; Rahmonov et al. 2021). The assumed faster organic matter mineralization in interrows can be also explained by its translocation from rows during ploughing and thus exposition of the material for oxidation, which can accelerate the process (Löf et al. 2012). Taking account of the above, it is difficult to explain why, on the contrary to other elements, pedogenic stocks of potassium strongly increased in interrows of AR between 2008 and 2020. Possibly, this could be explained by a decisive initial loss of this element in several initial years after the ploughing and the trees introducing. Then, a soil is weakly protected by trees, and O horizon is strongly exposed to denudation agents (e.g. wind), which primarily affect interrows being located higher than rows. This could involve especially high loss just of potassium, which is a very labile and susceptible to leaching element in a soil (Schlesinger 2021). In subsequent decades it could be expected that the differences in stocks of soil organic matter and pedogenic nutrients between rows and interrows of AR would continue declining. Finally, the micro-relief caused by ploughing disappears in the studied region after ca. 70 years after a soil scarification, however even after 100 years since ploughing, in a resultant flat area, lines of rows and interrows can be seen in a soil profile (Sewerniak et al. 2014).

## Conclusions

This study was conducted in a long-term research site located in one of the biggest post-fire areas in European temperate forests. The research was carried out using a 15-year timespan and was focused on the examinations of pedological and microclimatic variables as well as growth dynamics of trees regarding three variants of a management method used after fire. Our study delivers strong arguments to increase efforts in favour of the usage of natural regeneration with no soil preparation in forest restoration of post-fire areas in European temperate woodlands. High density of naturally regenerated stands involves efficient replenishment of soil organic matter and nutrients after the disturbance, which can be primarily linked to the more effective reconstruction of O horizon after fire than in the artificially regenerated site. Moreover, due to successful young trees protection against herbivore ungulates in extensive post-fire areas is treated as practically impossible (Miścicki et al. 2002), the high density of naturally regenerated young stands gives a chance to stay the core share of trees undisturbed by cervids to form vital mature stands in the future. Artificially regenerated plot with significantly lower tree density showed weaker restoration of soil organic matter and nutrients stocks in the studied period. The study suggests that artificial regeneration with subsequent soil preparation can be less favourable than natural forest recovery of a post-fire area with regard to the CO_2_ balance. For this issue, in further studies it would be interesting to also include carbon stocks allocated in trees to make the analysis regarding ecosystem storages of carbon more complex.

Traditionally, in temperate zone of Central Europe the role of fire in the functioning of woodlands has been marginalized (Tinner et al. 2005; Adámek et al. 2018). However, following a highlighted increasing risk of fire occurrence in forests of this zone (Schelhaas et al. 2003; Szczygieł et al. 2009), the further studies on examination of management methods in terms of sustainable restoration of post-fire temperate woodlands seem to be highly desirable.

### Supplementary information

Online Resource 1

Online Resource 2

Online Resource 3

Online Resource 4

## Data Availability

The processed data used in the study are available from the corresponding author by reasonable request.

## References

[CR1] Adámek M, Jankovská Z, Hadincová V, Kula E, Wild J (2018). Drivers of forest fire occurrence in the cultural landscape of Central Europe. Landsc Ecol.

[CR2] Aleksandrowicz-Trzcińska M, Drozdowski S, Brzeziecki B, Rutkowska P, Jabłońska B (2014). Effects of different methods of site preparation on natural regeneration of Pinus sylvestris in Eastern Poland. Dendrobiology.

[CR3] Barnett JP, Baker JB, Duryea ML, Dougherty PM (1991). Regeneration Methods. Forest Regeneration Manual. Forestry Sciences 36.

[CR4] Bednarek R, Dziadowiec H, Pokojska U, Prusinkiewicz Z (2004). Badania ekologiczno-gleboznawcze.

[CR5] Bolibok L, Andrzejczyk T (2008). Analysis of birch and pine seedling density in regeneration gaps on the basis of solar radiation model. Sylwan.

[CR6] Bullock EL, Woodcock CE, Olofsson P (2020) Monitoring tropical forest degradation using spectral unmixing and Landsat time series analysis. Remote Sens of Environ. 10.1016/j.rse.2018.11.011.

[CR7] Certini G (2005). Effects of fire on properties of forest soils: a review. Oecologia.

[CR8] Chaves Cardoso J, Burton PJ, Elkin CM (2020). A Disturbance Ecology Perspective on Silvicultural Site Preparation. Forests.

[CR9] Ciais P, Reichstein M, Viovy N (2005). Europe-wide reduction in primary productivity caused by the heat and drought in 2003. Nature.

[CR10] Crouzeilles R, Beyer HL, Monteiro LM, Feltran-Barbieri R, Pessôa ACM, Barros FSM, Lindenmayer DB, Lino EDSM, Grelle CEV, Chazdon RL, Matsumoto M, Rosa M, Latawiec AE, Strassburg BBN (2020) Achieving cost-effective landscape-scale forest restoration through targeted natural regeneration. Conserv Lett. 10.1111/conl.12709.

[CR11] DeBano LF, Neary DG, Ffolliott PF (1998). Fire’s Effects on Ecosystems.

[CR12] Dobrowolska D (2008). Natural regeneration on post-fire area in Rudy Raciborskie Forest District. Res Pap.

[CR13] Elgersma AM (1998). Primary forest succession on poor sandy soils as related to site factors. Biodivers Conserv.

[CR14] Fernandez-Anez N, Krasovskiy A, Müller M (2021). Current wildland fire patterns and challenges in Europe: A synthesis of national perspectives. Air Soil Water Res.

[CR15] Feurdean A, Tonkov S, Pfeiffer M, Panait A, Warren D, Vannière B, Marinova E (2019). Fire frequency and intensity associated with functional traits of dominant forest type in the Balkans during the Holocene. Eur J Res.

[CR16] Gray AN, Zald HS, Kern RA, North M (2005). Stand conditions associated with tree regeneration in Sierran mixed-conifer forests. Sci.

[CR17] Grüneberg E, Ziche D, Wellbrock N (2014). Organic carbon stocks and sequestration rates of forest soils in Germany. Glob Change Biol.

[CR18] Hammer Ø, Harper DAT, Ryan PD (2001). PAST: Paleontological statistics software package for education and data analysis. Palaeontol Electron.

[CR19] Hao B, Xu X, Wu F, Tan L (2022). Long-term effects of fire severity and climatic factors on post-forest-fire vegetation recovery. Forests.

[CR20] Hille M, Ouden J (2004). Improved recruitment and early growth of Scots pine (Pinus sylvestris L.) seedlings after fire and soil scarification. Eur J Res.

[CR21] Inbar A, Lado M, Sternberg M, Tenau H, Ben-Hur M (2014). Forest fire effects on soil chemical and physicochemical properties, infiltration, runoff, and erosion in a semiarid Mediterranean region. Geoderma.

[CR22] IUSS Working Group WRB (2022). World Reference Base for Soil Resources. International soil classification system for naming soils and creating legends for soil maps.

[CR23] Jahdi R, Salis M, Alcasena F, del Giudice L (2023) Assessing the Effectiveness of Silvicultural Treatments on Fire Behavior in the Hyrcanian Temperate Forests of Northern Iran. Env Manage (2023). 10.1007/s00267-023-01785-1. 10.1007/s00267-023-01785-1 36633631

[CR24] Jandl R, Lindner M, Vesterdal L, Bauwens B, Baritz R, Hagedorn F, Johnson DW, Minkkinen K, Byrne KA (2007). How strongly can forest management influence soil carbon sequestration. Geoderma.

[CR25] Jasińska J, Sewerniak P, Markiewicz M (2019). Links between slope aspect and rate of litter decomposition on inland dunes. Catena.

[CR26] Keeley JE (2009). Fire intensity, fire severity and burn severity: A brief review and suggested usage. Int J Wildl Fire.

[CR27] Kenk G, Guehne S (2001). Management of transformation in central Europe. Eco Manag.

[CR28] Kirdyanov AV, Saurer M, Siegwolf R, Knorre AA, Prokushkin AS, Churakova (Sidorova) OV, Fonti MV, Büntgen U (2020). Long-term ecological consequences of forest fires in the continuous permafrost zone of Siberia. Envir Res Lett.

[CR29] Kirschbaum MUF (2004). Soil respiration under prolonged soil warming: are rate reductions caused by acclimation or substrate loss?. Glob Change Biol.

[CR30] Knutti R, Sedláček J (2013). Robustness and uncertainties in the new CMIP5 climate model projections. Nat Clim Change.

[CR31] de las Heras J, Moya D, Vega JA, Daskalakou E, Vallejo VR, Grigoriadis N, Tsitsoni T, Baeza J, Valdecantos A, Fernández C, Espelta J, Fernandes P, Moreira F, Arianoutsou M, Corona P, De las Heras J (2012). Post-Fire Management of Serotinous Pine Forests. Post-Fire Management and Restoration of Southern European Forests. Managing Forest Ecosystems, 24.

[CR32] Lasslop G, Coppola AI, Voulgarakis A, Yue C, Veraverbeke S (2019). Influence of Fire on the Carbon Cycle and Climate. Curr Clim Change Rep..

[CR33] Lasslop G, Hantson S, Harrison SP, Bachelet D, Burton C, Forkel M, Forrest M, Li F, Melton JR, Yue C, Archibald S, Scheiter S, Arneth A, Hickler T, Sitch S (2020). Global ecosystems and fire: Multi-model assessment of fire-induced tree-cover and carbon storage reduction. Glob Change Biol.

[CR34] Li Y, Wang Z, Shi W, Yang H (2023). Litter quality modifies soil organic carbon mineralization in an ecological restoration area. Land Deg Dev.

[CR35] Löf M, Dey DC, Navarro RM, Jacobs DF (2012). Mechanical site preparation for forest restoration. New.

[CR36] Mallik A, Hu D (1997). Soil respiration following site preparation treatments in boreal mixedwood forest. Eco Manag.

[CR37] Marozas V, Racinskas J, Bartkevicius E (2007). Dynamics of ground vegetation after surface fires in hemiboreal Pinus sylvestris forests. Eco Manag.

[CR38] Masinda MM, Li F, Qi L, Sun L, Hu T (2022). Forest fire risk estimation in a typical temperate forest in Northeastern China using the Canadian forest fire weather index: case study in autumn 2019 and 2020. Nat Hazards.

[CR39] Mataix-Solera J, Cerdà A, Arcenegui V, Jordán A, Zavala LM (2011). Fire effects on soil aggregation: A review. Earth-Sci Rev.

[CR40] Mayer M, Prescott CE, Abaker WEA, Augusto L, Cécillon L, Ferreira GWD, James J, Jandl R, Katzensteiner K, Laclau J-P, Laganière J, Nouvellon Y, Paré D, Stanturf JA, Vanguelova EI, Vesterdal L (2020) Tamm Review: Influence of forest management activities on soil organic carbon stocks: A knowledge synthesis. For Eco Manage 10.1016/j.foreco.2020.118127.

[CR41] McDowell N, Pockman WT, Allen CD, Breshears DD, Cobb N, Kolb T, Plaut J, Sperry J, West A, Williams DG, Yepez EA (2008). Mechanisms of plant survival and mortality during drought: why do some plants survive while others succumb to drought?. N. Phytol.

[CR42] Meyer P, Ammer C, Wohlgemuth T, Jentsch A, Seidl R (2022). Forest Management. Disturbance Ecology. Landscape Series, vol 32.

[CR43] Miścicki S, Szerszenowicz A, Szerszenowicz K, Szukiel E (2002). The state of a post-fire forest regeneration and damage by cervids in the Rudy Raciborskie Forest District. Sylwan.

[CR44] Modrý M, Hubený D, Rejšek K (2004). Differential response of naturally regenerated European shade tolerant tree species to soil type and light availability. Eco Manag.

[CR45] Neary D, Klopatek CC, DeBano LF, Ffolliott PF (1999). Fire effects on belowground sustainability: a review and synthesis. Eco Manag.

[CR46] Niklasson M, Zin E, Zielonka T, Feijen M (2010). A 350-year tree-ring fire record from Bialowieza Primeval Forest, Poland: implications for Central European lowland fire history. J Ecol.

[CR47] O’Donnell JA, Romanovsky VE, Harden JW, McGuire AD (2009). The effect of moisture content on the thermal conductivity of moss and organic soil horizons from black spruce ecosystems in interior Alaska. Soil Sci.

[CR48] Pfeffer SE, Singh NJ, Cromsigt JPGM, Kalén C, Widemo F (2021) Predictors of browsing damage on commercial forests – A study linking nationwide management data, For Eco Manage 10.1016/j.foreco.2020.118597.

[CR49] R Core Team (2023). R: A language and environment for statistical computing.

[CR50] Rahmonov O, Skreczko S, Rahmonov M (2021). Changes in soil features and phytomass during vegetation succession in sandy Areas. Land.

[CR51] Ramantswana M, Guerra SPS, Ersson BT (2020). Advances in the mechanization of regenerating plantation forests: a review. Curr Rep..

[CR52] Schelhaas M-J, Nabuurs G-J, Schuck A (2003). Natural disturbances in the European forests in the 19th and 20th centuries. Glob Change Biol.

[CR53] Schlesinger WH (2021). Some thoughts on the biogeochemical cycling of potassium in terrestrial ecosystems. Biogeochem.

[CR54] Sewerniak P (2020). Plant species richness or soil fertility: which affects more the productivity of Scots pine in Central Europe?. Ann Res.

[CR55] Sewerniak P, Fifielska D, Bednarek R, Świtoniak M, Jankowski M, Bednarek R (2014). Przekształcenia morfologii i właściwości gleb na skutek zabiegów przygotowujących glebe do odnowienia drzewostanu. Antropogeniczne przekształcenia pokrywy glebowej Brodnickiego Parku Krajobrazowego.

[CR56] Sewerniak P, Gonet SS, Quaium M (2012). Impact of soil preparation method with rotary tiller on growth of Scots pine plants on poor sites of the Bydgoszcz Forest. Sylwan.

[CR57] Sewerniak P, Jankowski M (2021). Selected problems of sustainable management of rusty soils in forestry. Soil Sci Ann.

[CR58] Sewerniak P, Stelter P, Bednarek R (2017). Effect of site preparation method on dynamics of soil water conditions. Sylwan.

[CR59] Shakesby RA, Doerr SH (2006). Wildfire as a hydrological and geomorphological agent. Earth-Sci Rev.

[CR60] Skierucha W, Wilczek A, Alokhina O (2008). Calibration of a TDR probe for low soil water content measurements. Sens Actuat A-Phys.

[CR61] Statistics Poland (2022). Statistical Yearbook of Forestry.

[CR62] Szczygieł R, Ubysz B, Zawiła-Niedźwiecki T, Bytnerowicz A, Arbaugh M, Andersen Ch, Riebau A (2009). Spatial and Temporal Trends in Distribution of Forest Fires in Central and Eastern Europe. Wildland Fires and Air Pollution. Developments in Environmental Science 8.

[CR63] Tinner W, Conedera M, Ammann B, Lotter AF (2005). Fire ecology north and south of the Alps since the last ice age. Holoc.

[CR64] Tran BN, Tanase MA, Bennett LT, Aponte C (2020). High-severity wildfires in temperate Australian forests have increased in extent and aggregation in recent decades. PLoS ONE.

[CR65] Vincke C, Thiry Y (2008). Water table is a relevant source for water uptake by a Scots pine (Pinus sylvestris L.) stand: Evidences from continuous evapotranspiration and water table monitoring. Agr Meteor.

[CR66] Wang Y, Titus SJ, LeMay VM (1998). Relationships between tree slenderness coefficients and tree or stand characteristics for major species in boreal mixedwood forests. Can J Res.

[CR67] Wójcik G, Marciniak K, Andrzejewski L, Weckwerth P, Burak S (2006). Klimat. Toruń i jego okolice. Monografia przyrodnicza.

[CR68] Wojtkowiak R, Nowiński R, Tomczak RJ (2003). Soil temperature during wood slash burning. Sylwan.

[CR69] Wolf KD, Higuera PE, Davis KT, Dobrowski SZ (2021). Wildfire impacts on forest microclimate vary with biophysical context. Ecosph.

[CR70] Węgiel A, Jakubowski J, Molińska‑Glura M, Polowy K, Węgiel J, Gornowicz R (2023). Effect of logging residue removal and mechanical site preparation on productivity of the subsequent Scots pine (*Pinus sylvestris* L.) stands. Ann Sci.

[CR71] York RA, Battles JJ, Heald RC (2003). Edge effects in mixed conifer group selection openings: tree height response to resource gradients. Eco Manag.

[CR72] Yuan Y, Zhao Y, Gao Y, Gao G, Ren Y, Hou F (2022). The effect of tree species on soil organic carbon recovery in a restoration project is associated with vegetation biomass: Evidence from the Pingshuo Mine reclaimed ecosystem, North China. Land Deg Dev.

[CR73] Zell J, Hanewinkel M (2015). How treatment, storm events and changed climate affect productivity of temperate forests in SW Germany. Reg Env Change.

[CR74] Zerbe S (2002). Restoration of natural broad-leaved woodland in Central Europe on sites with coniferous forest plantations. Eco Manag.

[CR75] Zhang H, Zhou G, Wang Y, Tang C, Cai Y (2021) Clear-cut and forest regeneration increase soil N_2_O emission in Cunninghamia lanceolata plantations. Geodrema 401. 10.1016/j.geoderma.2021.115238.

[CR76] Zhao B, Zhuang Q, Shurpali N, Köster K, Berninger F, Pumpanen J (2021). North American boreal forests are a large carbon source due to wildfires from 1986 to 2016. Sci Rep..

