# Supplementary material for: Environmental effects of a management method used after fire on development of temperate Scots pine ecosystem: a 15-year study from Poland
Source: Environ Manage. 2023 Jun 9;72(5):978–90. doi: 10.1007/s00267-023-01843-8 (PMC10509113; doi:10.1007/s00267-023-01843-8)
Supplement: Supplementary file 3 — Online Resource 3 [file 267_2023_1843_MOESM3_ESM.docx]

**Environmental effects of a forest management method used after fire on development of temperate Scots pine ecosystem:
a 15-year study from Poland**

Sewerniak P.*, Markiewicz M., Tarnawska P., Wójcik M.

Environmental Management

*Corresponding author at: Department of Soil Science and Landscape Management, Nicolaus Copernicus University in Toruń, Lwowska 1,
87-100 Toruń, Poland, sewern@umk.pl (ORCID: 0000-0002-3071-3963)

**Online Resource 3.**

Table 1. Mean thickness (±SE) of topsoil horizons overlying the primary (pre-fire) A horizon in 2008 and 2020

| Horizon | NR | | NR-UC | | AR-r | | AR-ir | |
| --- | --- | --- | --- | --- | --- | --- | --- | --- |
|  | 2008 | 2020 | 2008 | 2020 | 2008 | 2020 | 2008 | 2020 |
| O | 3.8±0.8 | 4.2±0.3 | 2.7±0.4 | 4.0±0.4 | 1.7±0.2 | 2.0±0.3 | 0.7±0.1 | 1.5±0.4 |
| Aal | - | - | - | - | - | - | 5.3±0.4 | 8.4±0.4 |
| Obu | 2.0±0.2 | 0.9±0.1 | 2.5±0.5 | 1.1±0.2 | - | - | 3.5±0.9 | 0.9±0.1 |

Table 2. Topsoil mean stocks (±SE) of soil organic matter (OM), organic carbon (C), total nitrogen (N), and exchangeable Ca, Mg, K in 2008 and 2020.
NR – natural regeneration, NR-UC – natural regeneration under canopy, AR-r – artificial regeneration (rows), AR-ir – artificial regeneration (inter-rows).

| Horizon | NR | | NR-UC | | AR-r | | AR-ir | |
| --- | --- | --- | --- | --- | --- | --- | --- | --- |
|  | 2008 | 2020 | 2008 | 2020 | 2008 | 2020 | 2008 | 2020 |
| OM | | | | | | | | |
| O | 1.48±0.25 | 5.81±0.53 | 1.45±0.27 | 6.38±1.15 | 0.78±0.10 | 2.87±0.46 | 0.21±0.07 | 2.85±0.62 |
| Aal | - | - | - | - | - | - | 3.15±0.27 | 4.77±1.01 |
| Obu | 1.98±0.08 | 1.15±0.17 | 1.47±0.37 | 1.64±0.45 | - | - | 3.01±0.83 | 1.17±0.25 |
| A | 0.86±0.17 | 0.88±0.13 | 0.89±0.11 | 0.78±0.17 | 0.93±0.08 | 0.81±0.08 | 1.10±0.15 | 0.72±0.07 |
| C | | | | | | | | |
| O | 0.85±0.17 | 2.39±0.21 | 0.81±0.14 | 2.63±0.45 | 0.43±0.06 | 1.16±0.19 | 0.12±0.03 | 1.32±0.30 |
| Aal | - | - | - | - | - | - | 1.74±0.17 | 2.46±0.54 |
| Obu | 1.10±0.04 | 0.63±0.08 | 0.83±0.20 | 0.77±0.19 | - | - | 1.69±0.46 | 0.62±0.10 |
| A | 0.53±0.13 | 0.49±0.09 | 0.49±0.06 | 0.49±0.11 | 0.44±0.05 | 0.40±0.03 | 0.70±0.12 | 0.35±0.03 |
| N | | | | | | | | |
| O | 0.027±0.007 | 0.082±0.007 | 0.022±0.003 | 0.091±0.014 | 0.010±0.002 | 0.041±0.007 | 0.003±0.001 | 0.040±0.009 |
| Aal |  |  |  |  |  |  | 0.06±0.01 | 0.10±0.02 |
| Obu | 0.064±0.003 | 0.033±0.004 | 0.050±0.015 | 0.051±0.013 |  |  | 0.064±0.017 | 0.023±0.004 |
| A | 0.022±0.006 | 0.020±0.003 | 0.019±0.002 | 0.020±0.006 | 0.019±0.002 | 0.019±0.002 | 0.028±0.005 | 0.014±0.001 |
| Ca | | | | | | | | |
| O | 0.23 | 0.81±0.08 | 0.19 | 0.93±0.14 | 0.13 | 0.46±0.06 | 0.03 | 0.42±0.09 |
| Aal | - | - | - | - | - | - | 0.27 | 0.34±0.08 |
| Obu | 0.16 | 0.07±0.03 | 0.13 | 0.04±0.01 | - | - | 0.37 | 0.11±0.03 |
| A | 0.09 | 0.024±0.009 | 0.05 | 0.024±0.010 | 0.24 | 0.092±0.021 | 0.12 | 0.042±0.006 |
| Mg | | | | | | | | |
| O | 0.03 | 0.13±0.01 | 0.03 | 0.17±0.03 | 0.02 | 0.06±0.01 | 0.008 | 0.08±0.02 |
| Aal | - | - | - | - | - | - | 0.09 | 0.04±0.01 |
| Obu | 0.02 | 0.013±0.003 | 0.03 | 0.015±0.004 | - | - | 0.04 | 0.008±0.002 |
| A | 0.02 | 0.010±0.002 | 0.02 | 0.008±0.001 | 0.05 | 0.011±0.001 | 0.04 | 0.006±0.001 |
| K | | | | | | | | |
| O | 0.03 | 0.06±0.01 | 0.02 | 0.07±0.01 | 0.013 | 0.05±0.01 | 0.005 | 0.06±0.01 |
| Aal | - | - | - | - | - | - | 0.02 | 0.04±0.01 |
| Obu | 0.03 | 0.010±0.002 | 0.015 | 0.011±0.003 | - | - | 0.013 | 0.008±0.002 |
| A | 0.013 | 0.011±0.002 | 0.010 | 0.008±0.001 | 0.03 | 0.011±0.001 | 0.010 | 0.007±0.001 |
